# Influence of HLA Class I and II Polymorphisms on COVID-19 Severity in a South Brazilian Population

**DOI:** 10.3390/ijms26115341

**Published:** 2025-06-02

**Authors:** Sergio Grava, Matheus Braga, Victor Hugo de Souza, Afonso Carrasco Pepineli, Aléia Harumi Uchibaba Yamanaka, Christiane Maria Ayo, Joana Maira Valentini Zacarias, Andréa Name Colado Simão, Larissa Danielle Bahls Pinto, Quirino Alves de Lima Neto, Jeane Eliete Laguila Visentainer

**Affiliations:** 1 Post-Graduation Program in Biosciences and Physiopathology, Department of Clinical Analysis and Biomedicine, State University of Maringá, Maringá 87020-900, PR, Brazilmatheus.bragga@hotmail.com (M.B.); victoruem@gmail.com (V.H.d.S.); afonsocp@hotmail.com (A.C.P.); aleiayamanaka@hotmail.com (A.H.U.Y.); qalneto@uem.br (Q.A.d.L.N.); 2 Laboratory of Immunogenetics, Department of Dermatological, Infectious and Parasitic Diseases, Faculty of Medicine of São José do Rio Preto, São José do Rio Preto 15090-000, SP, Brazil; christiane.ayo@edu.famerp.br; 3 Department of Basic Chairs, Marília School of Medicine—FAMEMA, Marília 17519-030, SP, Brazil; 4 Department of Pathology, Clinical Analysis and Toxicology, State University of Londrina, Londrina 86038-350, PR, Brazil; deianame@yahoo.com.br; 5 Immunogenetics Laboratory, Department of Basic Health Sciences, State University of Maringá, Maringá 87020-900, PR, Brazil

**Keywords:** histocompatibility antigen classes, coronavirus, polymorphism, genetic, HLA-DR beta-chains, in silico model

## Abstract

The high variability of human leukocyte antigen (*HLA*) genes results in each molecule having distinct antigenic peptide binding capacities, potentially influencing the immune response to SARS-CoV-2. This study aimed to investigate associations between *HLA class I* (*A*, *B*) and *class II* (*DRB1*) polymorphisms and COVID-19 severity in a South Brazilian population, and to evaluate the binding affinity of alleles to viral peptides. A cross-sectional study included 503 unvaccinated patients with RT-qPCR-confirmed COVID-19: 145 non-severe, 129 severe, and 229 critical. *HLA* typing was performed using PCR-SSO and Luminex™ technology. The DRB1*11 allelic group was significantly associated with protection against severe and critical cases, while DRB1*15 was associated with increased risk; both remained significant after Bonferroni correction. Other allelic groups were associated with disease outcomes but lost significance after correction: B*49 and B*08 (risk); and B*37, B*50, and A*03 (protection). In silico analysis revealed that the DRB1*15 allele group showed a higher proportion of strong binders, mostly from non-structural proteins, while DRB1*11:01 binders, though fewer in number, were concentrated in the M protein. These results suggest functional differences in antigen presentation and reinforce the relevance of *class II HLA*, particularly *DRB1*, in modulating COVID-19 severity.

## 1. Introduction

The global outbreak of Coronavirus Disease 2019 (COVID-19), caused by Severe Acute Respiratory Syndrome Coronavirus 2 (SARS-CoV-2), has represented an unprecedented public health emergency [1]. The disease exhibits a broad range of clinical outcomes, from asymptomatic infection to critical illness and death [2]. While factors such as older age and chronic conditions, including cardiovascular disease and type 2 diabetes, are well-known predictors of disease severity and mortality, considerable variation exists among individuals and across populations [3,4]. These differences highlight the important role of immunological and genetic factors in the development of severe COVID-19 [5].

The immune system is a key determinant in shaping the host’s response to viral infections, influencing both the course and severity of disease [6]. Human leukocyte antigen (HLA) class I and class II molecules are central to initiating antigen-specific immune responses. These molecules are expressed on the surface of cells and present antigenic peptides to T lymphocytes. HLA class I molecules interact with cytotoxic CD8^+^ T cells, while class II molecules engage helper CD4^+^ T cells [7]. This mechanism of antigen presentation is essential for effective immune defense. Class I molecules facilitate the recognition and elimination of infected cells by CD8^+^ T cells, whereas class II molecules activate CD4^+^ T cells, which promote antibody production and regulate broader aspects of the immune response [8].

The extensive polymorphism of *HLA* genes leads to significant variation in the capacity of HLA molecules to bind antigenic peptides, which can influence the effectiveness of an individual’s immune response [9]. This variability may contribute to differences in susceptibility to infection and the severity of disease outcomes. In the context of COVID-19, such variation in peptide-binding affinity, driven by specific HLA alleles, may directly impact disease progression. Studies across different populations have identified distinct alleles associated with varying degrees of severity, suggesting a population-specific influence of HLA variants on clinical outcomes [10,11,12].

Given the influence of HLA molecules on immune response diversity, analyzing allele frequencies within specific populations can shed light on differential susceptibility and outcomes related to COVID-19. This study aimed to explore the relationship between polymorphisms in *HLA class I* (*A*, *B*) and *class II* (*DRB1*) genes and the severity of COVID-19 in a population from Southern Brazil. Additionally, the study assessed the predicted binding affinities between these alleles and SARS-CoV-2-derived peptides using in silico tools. By integrating genetic association analyses with functional computational predictions, this research provides a more integrated understanding of how HLA variability may affect disease progression. Moreover, it contributes original data from a genetically diverse population, enhancing the global perspective on COVID-19 immunogenetics.

## 2. Results

Significant associations were identified between patient characteristics and COVID-19 severity (categorized as non-severe, severe, and critical). Individuals over the age of 50 were more frequently observed in the severe and critical groups compared to the non-severe group. Male patients were also more commonly represented among the severe and critical cases. Moreover, conditions such as cardiovascular disease (CVD), type 2 diabetes mellitus, and tobacco use were more prevalent in patients with severe and critical disease, as shown in Table 1.

Among critically ill patients, significant differences were observed in age and the presence of CVD and type 2 diabetes mellitus when comparing survivors to non-survivors. Non-survivors tended to be older and showed a higher frequency of CVD and diabetes. No statistically significant differences were found in sex or tobacco-smoking status between the two groups, as presented in Table 2.

A relationship was observed between specific *HLA* allelic groups and the severity of COVID-19 (Table 3). The DRB1*11 allelic group was less common in severe and critical cases, suggesting a potential protective effect even after Bonferroni correction. The DRB1*15 allelic group was significantly more common in severe cases and severe + critical cases, suggesting an increased risk, even after Bonferroni correction in the non-severe vs. severe comparison. In the non-severe vs. critical comparison, the B*49 allelic group showed a risk association for critical cases, and in the non-severe vs. severe + critical comparison, the B*37 allelic group showed a protective association for severe + critical cases. When comparing severe vs. critical patients, the B*08 and B*50 allelic groups were associated with risk and protection for critical cases, respectively. In addition, in the survival vs. death comparison of critical patients, the A*03 allelic group was linked to a lower mortality risk.

The remaining allelic groups for all comparisons are represented in Supplementary Table S1. All *loci* were in agreement with Hardy–Weinberg equilibrium (HWE), except for *locus* A* in the non-severe group. The HWE comparison values are shown in Supplementary Table S2. The allelic groups DRB1*11 and DRB1*15, which were associated with COVID-19 severity in the non-severe vs. severe comparison, showed sufficient statistical power for the sample size, with 83% and 91% power, respectively.

Associations between HLA haplotypes and COVID-19 severity were also found (Table 4). The HLA-A*02~DRB1*11 haplotype was less frequent in the severe group, indicating a protective effect to severe cases when compared to non-severe vs. severe. Conversely, the HLA-A*02~DRB1*15 haplotype was identified as a risk factor for severe disease, particularly when compared to non-severe cases. Other haplotypes, such as HLA-A*11~B*35, A*24~DRB1*11, A*24~DRB1*13, A*24~DRB1*14, and A*18~DRB1*11, also showed significant associations, underscoring genetic contributions to disease severity when comparing non-severe vs. critical. When comparing non-severe vs. severe + critical, associations with HLA-A*11~B*35 and A*02~DRB1*15 remained. Even more curiously, the HLA-A*02~DRB1*11 was associated with risk for critical patients when compared to severe vs. critical.

Linkage disequilibrium (LD) was evaluated between the *HLA* gene pairs in individuals with different degrees of clinical severity (Supplementary Table S3). In non-severe cases, significant evidence of LD was observed for the *HLA-A~HLA-B* and *HLA-B~HLA-DRB1* pairs, while the *HLA-DRB1~HLA-A* pair showed no statistical significance. In severe cases, strong evidence of LD was found for the *HLA-B~HLA-DRB1* pair, while the *HLA-A~HLA-B* and *HLA-DRB1~HLA-A* pairs showed no significance. In critical cases, all analyzed pairs exhibited pronounced linkage disequilibrium, indicating a marked difference in this more severe clinical condition.

**Figure 1:**
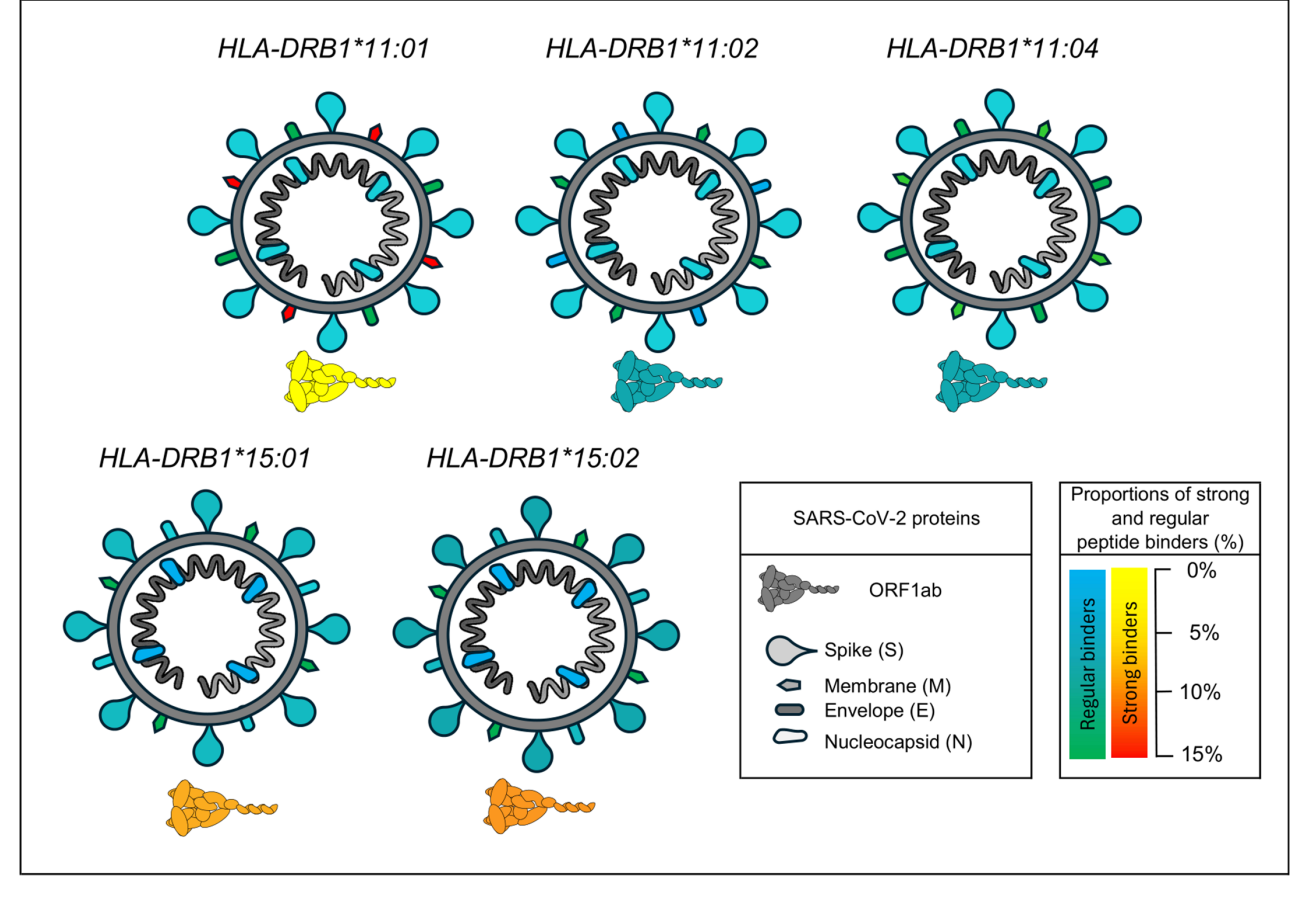
**Binding profile of SARS-CoV-2 protein-derived peptides to *HLA-DRB1* alleles.** Predicted frequency of strong binder peptides derived from SARS-CoV-2 non-structural (ORF1ab) and structural proteins (Spike, Membrane, Envelope, and Nucleocapsid) across six DRB1 alleles (DRB1*11:01, DRB1*11:02, DRB1*11:04, DRB1*15:01, and DRB1*15:02). Red, orange, and yellow colors indicate the presence of strong binders, representing different proportions of all predicted peptides: yellow (0–5%), orange (5.1–10%), and red (10.1–15%). Green and blue colors indicate the presence of regular binders only, meaning no strong binders were detected for those proteins in the respective alleles. The results highlight allele-specific differences in the ability of MHC class II molecules to present SARS-CoV-2-derived peptides.

The results of the in silico analysis showed that peptide sequences derived from SARS-CoV-2 viral proteins formed a higher proportion of strong and moderate binders with the DRB1*15 alleles group (6.8%) compared to DRB1*11 (5.5%). However, the binding proportions differed based on the specific viral protein and allele analyzed. For example, the DRB1*15:01 allele exhibited strong binders exclusively in non-structural proteins, which may influence the nature of the immune response. DRB1*11:01 contained a lower proportion of moderate binders (4.3%) than DRB1*11:02 (6.1%) and DRB1*11:04, but twice as many strong binders (0.4%) as the other two alleles combined (0.2%). Importantly, the strong binders for DRB1*11:01 were found in the M protein, while for the other, DRB1*11, allele groups were located in non-structural proteins (Figure 1).

## 3. Discussion

The Coronavirus Disease 2019 (COVID-19) pandemic has posed a major global health challenge, particularly among individuals with pre-existing conditions. Several host-related factors, including advanced age, male sex, and comorbidities such as cardiovascular disease and diabetes, have been consistently associated with worse outcomes [13]. In our cohort, these characteristics were significantly more frequent in severe and critical cases compared to non-severe cases (Table 1), confirming previously described risk profiles. While such clinical factors are well established, growing evidence points to the influence of genetic and immunological components in modulating COVID-19 severity. Among these, human leukocyte antigen (HLA) polymorphisms stand out as critical determinants of the host’s immune response to Severe Acute Respiratory Syndrome Coronavirus 2 (SARS-CoV-2), influencing disease progression and outcomes.

The interaction between SARS-CoV-2 and the host immune system, particularly the role of HLA molecules, is fundamental in initiating and modulating immune responses, thereby influencing the clinical progression of SARS-CoV-2 infection [9]. Numerous HLA alleles have been linked to both susceptibility to and severity of COVID-19; however, findings across studies have been inconsistent and, at times, contradictory. This variability suggests that the association between the HLA system and COVID-19 outcomes may be influenced by ethnic background, as HLA allele frequencies differ among populations [10,11,12]. These observations underscore the importance of investigating diverse geographic and genetic contexts, particularly within Brazil’s highly admixed population.

In this study, HLA-DRB1*11 allele was associated with a protective effect against severe and critical forms of COVID-19, and this association remained significant even after applying the Bonferroni correction. This allele has also been linked to COVID-19 outcomes in other studies. Consistent with our results, research conducted in Sardinia, Italy, reported that HLA-DRB1*11 was more frequently observed in non-severe cases compared to moderate and severe cases. However, in that study, the association lost statistical significance following Bonferroni correction [14]. Similarly, a study from Spain by Gutiérrez-Bautista et al. found that DRB1*11:01 was more common among healthy blood donors than in hospitalized COVID-19 patients, suggesting a potential protective role that also did not remain significant after correction [15].

A study conducted in Burkina Faso by Ouedraogo et al. reported that individuals carrying the HLA-DRB1*11 allele had an approximately sixfold increased risk of asymptomatic SARS-CoV-2 infection compared to those who tested negative for COVID-19 [16]. In contrast, research from Iran found a higher frequency of HLA-DRB1*11 in severe cases compared to mild ones, indicating a potential association with increased disease severity [17]. Another study from Iran, using high-resolution HLA typing, showed that HLA-DRB1*11:01 was significantly more prevalent in severe patients than in moderate cases, whereas HLA-DRB1*11:05 appeared more frequently in healthy individuals than in patients [18]. Additionally, a study from Poland identified DRB1*11:02 as being associated with a higher risk of symptomatic SARS-CoV-2 infection when compared to a control group of potential hematopoietic stem cell donors [19].

Our findings also revealed a significant association between the HLA-DRB1*15 allelic group and an increased risk of developing severe forms of COVID-19, with the association remaining significant after Bonferroni correction. Similar associations have been reported in various populations worldwide. In an Italian cohort, the HLA-DRB1*15:01 allele was significantly associated with severe and critical COVID-19 cases when compared to a control group of healthy individuals [20]. A study conducted in the United Arab Emirates likewise identified a positive association between HLA-DRB1*15:01 and disease severity [21]. In the United Kingdom, Poulton et al. reported an association between HLA-DRB1*15 and SARS-CoV-2 infection by comparing positive patients to published allele frequencies from a deceased donor population, although this association did not remain significant after Bonferroni correction [22].

Additional studies have also highlighted the possible involvement of the HLA-DRB1*15 allelic group in the severity of COVID-19. In India, Prasad et al. reported an association between HLA-DRB1*15 and both symptomatic infection and increased disease severity among renal transplant recipients [23]. Meanwhile, an Iranian study observed a lower frequency of HLA-DRB1*15 in COVID-19 patients compared to healthy controls; however, this allele group was more common in patients exhibiting lymphopenia, a condition frequently associated with severe and critical disease forms. These results suggest that despite some regional differences, HLA-DRB1*15 may act as a genetic marker linked to susceptibility to more severe COVID-19 outcomes [24].

HLA class II molecules, particularly those encoded by the *DRB1 locus*, are key players in the adaptive immune response to COVID-19. Low expression of HLA-DR on monocytes (mHLA-DR) has been consistently associated with immunosuppression and greater disease severity, especially in critically ill patients [25]. This reduction reflects impaired antigen presentation and has been linked to unfavorable outcomes. In our study, the allelic groups DRB1*11 and DRB1*15 showed significant associations with protection against and increased risk for severe COVID-19, respectively. These results are further supported by a recent meta-analysis of 28 studies involving over 13,000 individuals that also identified these alleles as important markers of disease severity, highlighting the critical role of HLA-DRB1 in COVID-19 progression [12].

Our in silico results suggest that differences in antigen presentation between *HLA-DRB1* alleles may influence the immune response to SARS-CoV-2. A higher proportion of strong and moderate binders was observed for the DRB1*15 allele group (6.8%) compared to DRB1*11 (5.5%). However, this apparent advantage does not seem to confer protection, as DRB1*15 was associated with increased severity in our cohort. This paradox suggests that the quality, timing, and immunological context of antigen presentation may be more important than binding affinity alone. In contrast, although the DRB1*11 allele group presented fewer total binders, the DRB1*11:01 variant showed strong binders targeting the M protein.

CD4^+^ T cell responses targeting structural proteins, such as the highly expressed M and N protein components of SARS-CoV-2, have been associated with more effective and durable immunity [26]. This may help explain the protective association observed for DRB1*11 in our study, as the presentation of epitopes from these proteins could promote a more coordinated immune response. Altogether, our findings reinforce the idea that not only the number of presented peptides, but which viral proteins are targeted, and in what immunological context, plays a critical role in determining disease outcomes. The presentation of non-structural protein epitopes by DRB1*15, for example, might contribute to a dysregulated or insufficient immune response, as has been proposed in studies exploring immune pathology in severe COVID-19. Integrating clinical, immunological, and computational evidence is essential to fully understand the role of HLA in modulating COVID-19 severity and can inform future personalized immunotherapy or vaccine strategies.

In our study, two additional *class I* alleles group were associated with COVID-19 severity: HLA-B*37 emerged as a potential protective factor against severe and critical cases, whereas HLA-B*49 was identified as a possible risk factor specifically for critical cases. However, both associations lost statistical significance following Bonferroni correction. The relationship between these allele groups and COVID-19 severity remains poorly explored in the literature, underscoring a gap in targeted investigations of these markers. A study conducted in Turkey reported an association between HLA-B*49 and increased susceptibility to SARS-CoV-2 infection among patients receiving renal replacement therapy [27]. Similarly, research from Russia identified HLA-B*49 as a predisposing factor for infection with the Delta variant (B.1.617.2) [28]. Conversely, an ecological study in Italy found an inverse log-linear correlation between HLA-B*49 and COVID-19 incidence during the peak of the national outbreak in April 2020; however, this correlation lost significance after adjustment for confounding variables through multiple regression analysis [29]. To date, no studies have reported any association between HLA-B*37 and either susceptibility to or severity of COVID-19.

In the comparative analysis between severe and critical COVID-19 patients, two HLA class I allelic groups initially showed associations: HLA-B*08 appeared to act as a potential protective factor against progression to critical illness, while HLA-B*50 was associated with an increased risk. However, both associations lost statistical significance after Bonferroni correction. Supporting our observations, HLA-B*50 has been previously linked to disease severity. In a Romanian population, Vică et al. reported an association between B*50 and greater susceptibility to severe COVID-19 compared to mild cases [30]. Likewise, a study conducted in Saudi Arabia found that the allele HLA-B*50:01:01G was associated with an elevated risk of severe disease when compared to healthy controls [31]. In contrast, the role of HLA-B*08 remains more controversial. Contrary to our findings, several ecological studies have reported a positive correlation between B*08 and increased COVID-19-related mortality, based on comparisons of national mortality rates and allele frequencies from public databases [32,33]. An Italian ecological study using national-level data also identified a positive correlation between B*08 frequency and COVID-19 incidence during the peak of the outbreak [29].

Our analysis of outcomes within the critical patient group suggests that the HLA-A*03 allele may be associated with a reduced risk of mortality. Consistent with this finding, Vică et al. conducted a study in Romania indicating that individuals carrying HLA-A*03 were less prone to developing extremely severe manifestations of COVID-19 [30]. Similarly, a Russian investigation found that an HLA class I-based risk score was notably elevated in younger adults who died of the disease compared to older individuals, with the presence of HLA-A*03:01 correlating with a lower risk profile [34]. On the other hand, contrasting evidence has emerged from other regions. Research in Iran identified HLA-A*03 as a possible contributor to susceptibility to SARS-CoV-2 infection [35], while a study from Saudi Arabia observed a higher prevalence of this allele among individuals who died from COVID-19 compared to survivors [36]. These discrepancies suggest that the role of HLA-A*03 in COVID-19 outcomes may be influenced by population-specific genetic backgrounds and environmental contexts.

Our haplotype analysis indicates that DRB1*11, together with A*02, A*24, and B*18, may contribute to resistance against the progression to severe or critical forms of COVID-19. Supporting this, a study conducted in Italy identified the haplotype HLA-A*02:01~B*18:01~C*07:01~DRB1*11:04 as significantly and negatively correlated with both disease severity and fatal outcomes [37], thus reinforcing the idea that DRB1*11, when occurring alongside A*02 and B*18, could play a role in limiting disease progression. Interestingly, in our dataset, the haplotype A*02~DRB1*11 appeared more frequently among critical patients when compared to those with severe disease, suggesting that its protective effect might be more relevant during earlier stages of infection or viral exposure. Several additional HLA combinations also demonstrated notable associations with disease severity in our cohort. Haplotypes such as A*02~DRB1*15, A*11~B*35, A*24~DRB1*11, A*24~DRB1*13, and A*24~DRB1*14 showed significance, particularly in comparisons between non-severe and critical groups, or between non-severe versus combined severe/critical cases. However, these particular haplotype associations have not yet been documented in the literature, underscoring a current gap in scientific understanding and emphasizing the exploratory nature of our findings.

The presence of linkage disequilibrium between *HLA loci* across different severity groups may partially explain these associations. In more severe cases, particularly among critically ill patients, all analyzed gene pairs exhibited significant linkage disequilibrium. This suggests that the observed effect of a haplotype may be driven by a specific allele inherited together with another, which may represent the true risk or protective factor. It is also important to consider the methodological limitations inherent to haplotype analysis in this study. The haplotypes were computationally inferred rather than directly determined through genotyping, which may introduce uncertainty. Furthermore, the relatively small sample sizes within each clinical subgroup could reduce the statistical robustness of the associations observed and limit the broader applicability of these results.

The genotyping results for the HLA *loci* generally conformed to Hardy–Weinberg equilibrium, with the exception of the A* *locus* in the non-severe patient group. It is worth emphasizing that our cohort consists exclusively of COVID-19 patients rather than a healthy control population, which may influence allele frequency distributions. This particular sample might reflect specific genetic characteristics shaped by selective pressures exerted by the virus, possibly explaining the deviation observed at the A* *locus*. Although this departure from equilibrium does not undermine the validity of our analysis, it suggests that genetic factors could be modulating the immune response in distinct ways, potentially affecting disease severity, especially when comparing non-severe cases to asymptomatic individuals. A notable limitation of our study is the absence of an asymptomatic group, which would have provided valuable insight into whether the observed genetic variation is truly linked to severity or influences immune responses through other mechanisms. Another limitation involves the lack of data on SARS-CoV-2 genetic variants in our cohort. Viral strains differ in their mutation profiles, which can alter immune evasion strategies and clinical outcomes, possibly interacting with host genetics such as HLA types. Nevertheless, it is important to note that the study period (March 2020 to April 2021) coincided predominantly with the circulation of the Gamma variant (P.1) in the region [38,39], providing context for interpreting our findings.

Variability in reported connections between HLA alleles and COVID-19 across studies is influenced not only by population ethnicity but also by differences in research design. Ecological studies, for instance, have important limitations, including the use of aggregated population data and the lack of individual-level control for clinical and demographic variables, which can lead to misleading interpretations. Furthermore, many available studies compare broad groups, such as infected individuals versus healthy controls, without stratifying disease severity, and they often involve small sample sizes, thus limiting the statistical power and generalizability of their findings. In our study, we sought to overcome these limitations. Patients were carefully classified based on the World Health Organization criteria for disease severity, ensuring standardized and comparable groupings. Our sample size was adequate and provided sufficient statistical power to detect relevant associations. In addition, we employed robust statistical analyses, including logistic regression to adjust for potential confounders, and applied Bonferroni correction to control for type I error. These methodological strengths contribute to the consistency and scientific rigor of our findings.

## 4. Materials and Methods

### 4.1. Study Population

This cross-sectional study was conducted prior to the introduction of COVID-19 vaccination and included patients diagnosed by Reverse Transcriptase Quantitative Polymerase Chain Reaction (RT-qPCR). Participants were recruited between May 2020 and April 2021 from the University Hospital of Londrina at the State University of Londrina and from Hospital Paraná in Maringá, both located in Southern Brazil. The study was approved by the Ethics Committees of the Unicesumar—The University Center of Maringa (CAAE: 37322420.4.0000.5539), approved on 10 September 2020) and the State University of Londrina (CAAE: 31656420.0.0000.5231, approved on 27 May 2020). All participants, or their legal representatives in the case of critically ill patients, provided informed consent after being clearly informed about the objectives and procedures of the study.

**Figure 2:**
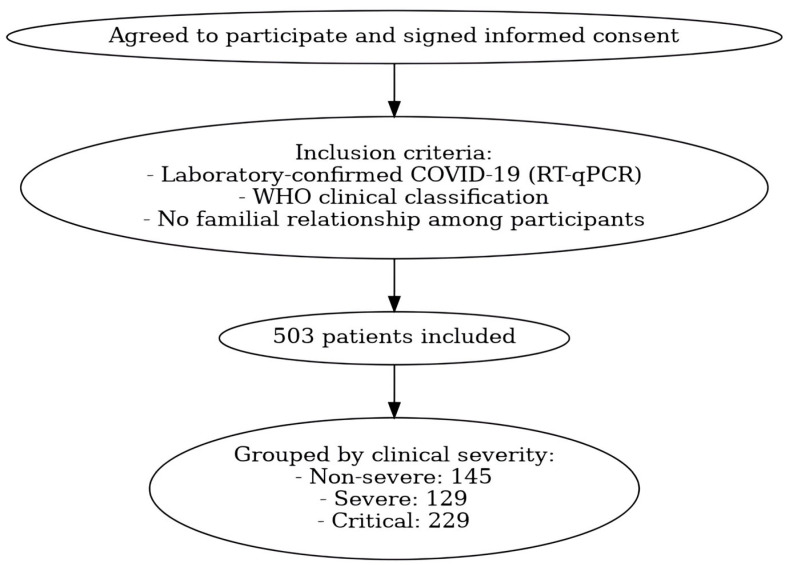
Patient selection flowchart. Flowchart illustrating the inclusion process and distribution of unvaccinated patients diagnosed with COVID-19, according to World Health Organization (WHO) severity classification.

A total of 503 unvaccinated patients were included, following the criteria of laboratory-confirmed COVID-19 diagnosis, classification according to the World Health Organization (WHO) [40], and no familial relationship among participants. Patients were categorized into non-severe (*n* = 145), severe (*n* = 129), and critical (*n* = 229) groups. A summary of the patient selection and classification process is illustrated in Figure 2.

Non-severe COVID-19 was defined by the absence of any severe or critical features. Severe cases were characterized by oxygen saturation below 90% on room air, accompanied by clinical signs of pneumonia or respiratory distress. Critical cases included individuals with acute respiratory distress syndrome, sepsis, or septic shock, or those requiring advanced life support, including mechanical ventilation or vasopressor therapy. All participants were classified as being from a mixed ethnic background, reflecting the demographic composition of Paraná state, with a predominance of European ancestry (80.6%), followed by African (12.5%) and Amerindian (7.0%) origins [41].

### 4.2. Sample Collection and DNA Extraction

Following the patients’ admission, peripheral blood samples were gathered in tubes containing ethylenediaminetetraacetic acid (EDTA) anticoagulant and dispatched to the Laboratório de Imunogenética—UEM. Genomic DNA was isolated using the BIOPUR^®^ extraction kit (Biometrix, Curitiba, Brazil), following the supplier’s protocol. The concentration of the extracted material was evaluated by measuring the optical density using a Nanodrop 2000^®^ spectrophotometer (Wilmington, DE, USA).

### 4.3. HLA Genotyping

Human leukocyte antigen (HLA), *HLA-A*, *HLA-B*, and *HLA-DRB1* were genotyped using Polymerase Chain Reaction with Sequence-Specific Oligonucleotide Probe (PCR-SSO) protocols based on Luminex™ technology. The genotyping was performed with the LABType™ CWD kit (One Lambda Inc., Canoga Park, CA, USA) at low-to-medium resolution, following the manufacturer’s instructions. In this method, genomic DNA was initially amplified by PCR using biotinylated sequence-specific primers. The resulting PCR products were then hybridized with oligonucleotide probes bound to fluorescent microspheres, which were labeled with R-phycoerythrin-conjugated streptavidin (SAPE). Fluorescence intensity was measured via flow cytometry using the FLEXMAP 3D™ system, and the results were analyzed using HLA Fusion software, version 4.6 (One Lambda Inc., Canoga Park, CA, USA).

### 4.4. In Silico Analysis

The in silico analyses in this study were conducted using a script developed in the R environment for statistical computing (version 4.2.0, R Foundation for Statistical Computing, Vienna, Austria) to automate the process of obtaining binding predictions from the NetMHCpan tool through its API. The input data, in FASTA format, included the amino acid sequences of SARS-CoV-2 proteins (NCBI Reference Sequence: NC_045512.2). The NetMHCpan algorithm versions used were 4.0 for MHC class I and 4.1 for MHC class II [42]. The predicted binding affinity was reported as IC50 values (nM), representing the concentration needed to inhibit 50% of binding. Peptides of length 9 and 13 amino acids were used for MHC-I and MHC-II predictions, respectively. Peptides were then categorized by binding strength: for MHC-I, strong binders (IC50 < 50 nM), intermediate binders (50–500 nM), weak binders (500–5000 nM), and non-binders (>5000 nM); for MHC-II, strong binders (IC50 < 50 nM), intermediate binders (50–1000 nM), weak binders (1000–5000 nM), and non-binders (≥5000 nM). The results were summarized in bar charts, showing the percentage distribution of peptide binding affinities for each SARS-CoV-2 protein and for the complete proteome.

### 4.5. Statistical Analysis

Hardy–Weinberg equilibrium and linkage disequilibrium were assessed using Arlequin (version 3.5.2.2, University of Bern, Bern, Switzerland) [43]. QUANTO (version 1.2.4, University of Southern California, Los Angeles, CA, USA) software was used to calculate the statistical power of the associated alleles using the following parameters: unmatched case control, gene only, population allele frequency, dominant model, population risk (0.05%), number of patients, and odds ratio values [44]. The remaining statistical analyses were executed using the R environment for statistical computing (version 4.2.1, R Foundation for Statistical Computing, Vienna, Austria) [45], employing specific packages. Clinical characteristics of patients, such as age, sex, smoking, and comorbidities, were analyzed using the “stats” package (R base). Continuous variables were compared using Student’s *t*-tests, while categorical variables were analyzed using chi-square tests.

Association analyses between *HLA* allelic groups and COVID-19 severity were carried out using a generalized linear model with logistic regression implemented in The “midasHLA” package [46]. Adjustments for potential confounders, including age, sex, and comorbidities, were included in the regression models. Bonferroni adjustment was applied manually to *p*-values to correct for multiple hypothesis testing. Haplotype analyses were further supported by “BIGDAWN” package [47], using expectation–maximization, facilitating the visualization and comprehensive analysis of multilocus genetic associations. A significance level of 0.05 was adopted, and *p*-values below this threshold were interpreted as statistically significant. The results were interpreted considering the biological relevance of the findings and the robustness of statistical models applied.

## 5. Conclusions

A significant link was identified between HLA class II (*DRB1*) allele groups and COVID-19 severity in a South Brazilian population. After Bonferroni correction, the DRB1*11 allelic group remained associated with protection against severe and critical cases, while DRB1*15 was linked to an increased risk of severity, independent of age, sex, smoking, cardiovascular diseases, and type 2 diabetes mellitus. In silico analysis revealed that the DRB1*15 allele group showed a higher proportion of strong binders, mostly from non-structural proteins, while DRB1*11:01 binders, though fewer, were concentrated in the M protein.

## Figures and Tables

**Table 1:** Summary of clinical and epidemiological characteristics across COVID-19 severity groups.

Characteristics	Non-Severe N: 145	Severe N: 129	Critical N: 229	*p*-Value ^a^	*p*-Value ^b^	*p*-Value ^c^
Age						
Mean ± SD	46.74 ± 14.41	60.58 ± 16.20	65.02±16.60	<0.01 ^d^	<0.01 ^d^	0.01 ^d^
≤50	79 (54%)	40 (31%)	44 (24%)	<0.01 ^e^	<0.01 ^e^	0.01 ^e^
>50	66 (46%)	89 (69%)	185 (76%)			
Sex						
Female	90 (62%)	62 (48%)	81 (35%)	0.02 ^e^	<0.01 ^e^	0.02 ^e^
Male	55 (38%)	67 (52%)	148 (65%)			
CVD						
No	117 (81%)	62 (40%)	95 (41%)	<0.01 ^e^	<0.01 ^e^	0.27 ^e^
Yes	28 (19%)	67 (60%)	134 (59%)			
Diabetes						
No	134 (92%)	99 (77%)	146 (64%)	<0.01 ^e^	<0.01 ^e^	0.01 ^e^
Yes	11 (8%)	30 (23%)	83 (36%)			
Tobacco smoking				<0.01 ^e^	<0.01 ^e^	0.4 ^e^
No	140 (97%)	106 (82%)	180 (79%)			
Yes	5 (3%)	23 (18%)	49 (21%)			

Abbreviations: SD, standard deviation; CVD, cardiovascular disease; Diabetes: type 2 diabetes mellitus; ^a^ non-severe vs. severe; ^b^ non-severe vs. critical; ^c^ severe vs. critical; ^d^ comparison between groups was conducted using Student’s *t*-test; ^e^ differences between categorical variables were assessed using a chi-square (χ^2^) test.

**Table 2:** Outcome characteristics in patients with critical forms of COVID-19.

Characteristics	Survival—N: 115	Death—N: 114	*p*-Value
Age			
Mean ± SD	58.11 ± 15.29	72 ± 14.92	<0.01 ^a^
≤50	32 (28%)	12 (11%)	<0.01 ^b^
>50	83 (72%)	102 (89%)	
Sex			
Female	39 (34%)	42 (37%)	0.70 ^b^
Male	76 (66%)	72 (63%)	
CVD			
No	57 (50%)	38 (33%)	0.01 ^b^
Yes	58 (50%)	76 (67%)	
Diabetes			
No	86 (75%)	60 (53%)	<0.01 ^b^
Yes	29 (25%)	54 (47%)	
Tobacco smoking			
No	87 (76%)	93 (82%)	0.30
Yes	28 (24%)	21 (18%)	

Abbreviations: SD, standard deviation; CVD, cardiovascular disease; Diabetes, type 2 diabetes mellitus; ^a^ comparison between groups was conducted using Student’s *t*-test; ^b^ differences between categorical variables were assessed using a chi-square (χ^2^) test.

**Table 3:** Human leukocyte antigen allelic groups are associated with the severity of COVID-19 independent of age, sex, cardiovascular diseases, type 2 diabetes mellitus, and tobacco smoking.

**Non-Severe (145) vs. Severe (129)**					
Allele	Non-Severe N (%)	Severe N (%)	OR (CI)	*p*-value	Bonferroni
DRB1*11	55 (18.97%)	27 (10.47%)	0.37 (0.20–0.68)	<0.01	0.03
DRB1*15	20 (6.90%)	38 (14.73%)	2.80 (1.43–5.59)	<0.01	0.04
**Non-Severe (145) vs. Critical (229)**					
Allele	Non-Severe N (%)	Critical N (%)	OR (CI)	*p*-value	Bonferroni
B*49	4 (1.38%)	15 (3.28%)	3.79 (1.13–15.33)	0.04	1.00
DRB1*11	55 (18.97%)	54 (11.79%)	0.51 (0.29–0.88)	0.01	0.13
**Non-Severe (145) vs. Severe + Critical (358)**					
Allele	Non-Severe N (%)	Severe + Critical N (%)	OR (CI)	*p*-value	Bonferroni
B*37	5 (1.72%)	4 (0.56%)	0.16 (0.03–0.83)	0.02	1.00
DRB1*11	55 (18.97%)	81 (11.31%)	0.46 (0.28–0.75)	<0.01	0.04
DRB1*15	20 (6.90%)	91 (12.71%)	1.88 (1.05–3.45)	0.03	0.39
**Severe (129) vs. Critical (229)**					
Allele	Severe N (%)	Critical N (%)	OR (CI)	*p*-value	Bonferroni
B*08	18 (6.98%)	13 (2.84 %)	0.40 (0.18–0.87)	0.02	0.31
B*50	2 (0.78%)	17 (3.71 %)	6.28 (1.67–41.26)	0.01	0.62
**Critical-Survival (115) vs. Death (114)**					
Allele	Survival N (%)	Death N (%)	OR (CI)	*p*-value	Bonferroni
A*03	28 (12.17%)	15 (6.58%)	0.37 (0.17–0.77)	0.01	0.19

Abbreviations: N, total number of alleles; (%), proportion of alleles; OR, odds ratio; CI, confidence interval; *p*-value, statistical analysis was performed by logistic regression; Bonferroni, *p*-value multiplied by the number of alleles at each *locus*.

**Table 4:** Association of human leukocyte antigen haplotypes with clinical severity in COVID-19 patients.

**Non-Severe (145) vs. Severe (129)**				
**Haplotype**	**Non-Severe N (%)**	**Severe N (%)**	**OR (CI)**	***p*-Value**
A*02~DRB1*11	18 (6.20%)	2 (0.77%)	0.12 (0.01–0.50)	<0.01
A*02~DRB1*15	2 (0.68%)	15 (5.81%)	8.89 (2.03–80.61)	<0.01
**Non-Severe (145) vs. Critical (229)**				
**Haplotype**	**Non-Severe N (%)**	**Critical N (%)**	**OR (CI)**	***p*-Value**
A*11~B*35	10 (3.44%)	3 (0.65%)	0.18 (0.03–0.73)	<0.01
A*24~DRB1*11	9 (3.10%)	2 (0.43%)	0.14 (0.01–0.67)	<0.01
A*24~DRB1*13	8 (2.75%)	3 (0.65%)	0.23 (0.04–0.98)	0.01
A*24~DRB1*14	1 (0.34%)	11 (2.40%)	7.11 (1.02–306.76)	0.02
B*18~DRB1*11	8 (2.75%)	3 (0.65%)	0.23 (0.04–0.98)	0.01
**Non-Severe (145) vs. Severe + Critical (358)**				
**Haplotype**	**Non-Severe N (%)**	**Severe + Critical N (%)**	**OR (CI)**	***p*-Value**
A*11~B*35	11 (3.79%)	9 (1.25%)	0.32 (0.12–0.87)	<0.01
A*02~DRB1*15	3 (1.03%)	29 (4.05%)	4.04 (1.24–20.86)	0.01
**Severe (129) vs. Critical (229)**				
**Haplotype**	**Severe N (%)**	**Critical N (%)**	**OR (CI)**	***p*-Value**
A*02~DRB1*11	3 (1.16%)	21 (4.58%)	4.08 (1.20–21.56)	0.01

Abbreviations: N, total number of haplotypes; (%), proportion of haplotype; OR, odds ratio; CI, confidence interval; *p*-value, statistical analysis was performed by Pearson’s χ^2^-test.

## Data Availability

All relevant data generated or analyzed during this study are included in this published article and its Supplementary Material.

## Supplementary Materials

The following supporting information can be downloaded at https://www.mdpi.com/article/10.3390/ijms26115341/s1.
