# Supplementary material for: Influence of HLA Class I and II Polymorphisms on COVID-19 Severity in a South Brazilian Population
Source: Int J Mol Sci. 2025 Jun 2;26(11):5341. doi: 10.3390/ijms26115341 (PMC12154496; doi:10.3390/ijms26115341)
Supplement: Supplementary file 1 [file ijms-26-05341-s001.zip › Supplementary Tables 2 and 3.pdf]

Table S2. Hardy-Weinberg equilibrium for *HLA-A*, *HLA-B* and *HLA-DRB1*, in the non-severe, severe and critical patients with COVID-19.

	**Obs. Het.**[a]	**Exp. Het.**[b]	**P-value**[c]
**Non Severe**			
HLA-A	0.81	0.88	**0.01**
HLA-B	0.92	0.93	0.21
HLA-DRB1	0.91	0.88	0.22
**Severe**			
HLA-A	0.89	0.90	0.10
HLA-B	0.93	0.92	0.07
HLA-DRB1	0.84	0.89	0.29
**Critical**			
HLA-A	0.90	0.87	0.05
HLA-B	0.91	0.93	0.24
HLA-DRB1	0.87	0.89	0.34

Abbreviations: [a]Observed heterozygosity index; [b] Expected heterozygosity index under Hardy-Weinberg equilibrium (HWE); [c]Probability values for Guo and Thompson HWE tests.

Table S3. Association between linkage disequilibrium and different degrees of severity.

	LnLHood LD[a]	LnLHood LE[b]	Exact P=[c]
**Non Severe**			
HLA-A~HLA-B	-1194.62106	-1385.84847	0.00
HLA-B~HLA-DRB1	-1166.66922	-1329.2978	0.00
HLA-DRB1~HLA-A	-1102.41235	-1189.39181	0.29
**Severe**			
HLA-A~HLA-B	-1067.10245	-1217.09383	0.28
HLA-B~HLA-DRB1	-1007.86006	-1169.27253	0.00
HLA-DRB1~HLA-A	-1005.28580	-1098.26903	0.44
**Critical**			
HLA-A~HLA-B	-1936.43465	-2171.54591	0.00
HLA-B~HLA-DRB1	-1954.59501	-2146.43112	0.00
HLA-DRB1~HLA-A	-1789.85291	-1910.57721	0.00

Abbreviations: [a] Logarithm of the Likelihood under the Linkage Disequilibrium Hypothesis; Linkage Equilibrium Hypothesis; [c] values for the analyzed HLA gene pairs, with Exact P values indicating statistical significance
